# Prognostic value of pretreatment procalcitonin and neutrophil–lymphocyte ratio in extensive-stage small-cell lung cancer

**DOI:** 10.1080/15384047.2024.2331273

**Published:** 2024-03-27

**Authors:** Dongfang Chen, Jianlin Xu, Yizhuo Zhao, Baohui Han, Runbo Zhong

**Affiliations:** Department of Respiratory and Critical Care Medicine, Shanghai Chest Hospital, Shanghai Jiao Tong University, School of Medicine, Shanghai, China

**Keywords:** Extensive-stage small-cell lung cancer, neutrophil-to-lymphocyte ratio, procalcitonin, progression-free survival

## Abstract

**Background:**

To investigate the influence of pretreatment neutrophil-to-lymphocyte ratio (NLR) and procalcitonin (PCT) on progression-free survival (PFS) in extensive-stage small-cell lung cancer (SCLC) patients.

**Method:**

A total of 100 extensive-stage SCLC patients were enrolled in our study. Patients were stratified according to the median values of pretreatment NLR and PCT levels: low NLR group (NLR ≤3.17), high NLR group (NLR＞3.17), low PCT group (PCT ≤0.06; ng/ml), high PCT group (PCT＞0.06; ng/ml). The Kaplan-Meier method and multivariable Cox regression model were used to reveal the prognostic effects of pretreatment NLR and PCT on PFS.

**Results:**

The median PFS of the total extensive-stage SCLC patients was 6.0 months. The median PFS of low pretreatment NLR group (NLR ≤3.17) was not significantly different from that of high pretreatment NLR group (6.2 months vs 5.8 months; *p* = .675). Patients with low pretreatment PCT (PCT ≤0.06; ng/ml) had significantly better PFS than patients with high pretreatment PCT (PCT＞0.06; ng/ml) (6.9 months vs 5.7 months; *p* = .043). With the multivariable Cox regression analysis, the response to first-line chemotherapy (*p* ≤ .001) and pretreatment PCT (HR = 0.516; 95%CI 0.326–0.817; *p* = .005) were identified as independent factors associated with PFS.

**Conclusion:**

Pretreatment PCT is an independent factor associated with PFS in extensive-stage SCLC patients treated with first-line chemotherapy, but pretreatment NLR reflects no significant prognostic value in our study.

## Introduction

SCLC accounts for about 15% −20% of lung cancer cases.^1^ SCLC belongs to one of neuroendocrine tumors, which is characterized by rapid tumor proliferation, tendency for early and extensive metastases, and poor prognosis.^1^ About 60% of the SCLC patients are in an extensive stage at the time of diagnosis.^2^ The first-line standard treatment for extensive-stage SCLC patients mainly remains chemotherapy with platinum-etoposide. The responses of posterior-line treatments for extensive-stage SCLC patients are limited and poor, and the median overall survival(OS) is approximately 8 to 12 months.^3^

With the deep understanding of tumor microenvironment, it has been gradually clear that there is a complex relationship among inflammation, immunity, and cancer.^4^ Inflammation has impacts on almost each step of tumorigenesis including tumor initiation, tumor promotion, and metastatic progression.^4^ And immune surveillance plays an important role in preventing or inhibiting tumor growth.^4^ Biochemical markers of inflammatory response have been incorporated in prognostic scores for several types of cancer. Lymphocytes, especially T cells are commonly found within the tumor microenvironment, which mainly engage in anti-tumor immune responses. NLR, a typical marker of inflammation and immune status has been associated with prognosis in various malignant tumors such as gastric cancers, colorectal cancers, pancreatic cancers, and even lung cancers.^5^ A higher pretreatment NLR generally predicts poorer prognosis, but it remains unclear whether pretreatment NLR has an influence on the short-term survival of extensive-stage SCLC patients.

Serum PCT as a biomarker has been developed over the past two decades for the diagnosis of bacterial infections, including sepsis, community acquired acute pneumonia and exacerbation of chronic obstructive pulmonary diseases (COPD).^6^ PCT-based strategy is widely used to guide antibiotic therapy clinically. PCT can, however, be elevated in some neoplastic situations while there is no infectious process, particularly in tumorous diseases like medullary thyroid cancer and SCLC, in which PCT is secreted by the tumor cells.^7^ It has also been reported that SCLC cell lines could produce PCT in vitro.^8^ In a study reported by Walter et al., elevated PCT was present in medullary thyroid cancer patients without infection, besides, the survival analysis indicated that elevated PCT was associated with poor prognosis.^9^ Patout et al. reported that higher PCT levels were detected in the tumors with neuroendocrine component, for example, PCT levels were significantly higher in SCLCs than in pulmonary adenocarcinomas.^10^ A case report also described a phenomenon that the PCT levels were relatively high in a patient with metastatic SCLC but without infection during admission, and surprisingly the PCT levels appeared a trend of decrease after anti-cancer treatment.^11^ As we observed in clinical practice, the elevation of PCT was present in some small-cell carcinoma cases, as well as in large-cell carcinomas with neuroendocrine component. However, few studies reveal the implications of PCT in SCLC patients, especially the prognostic value of PCT.

Thus, the aim of our retrospective study is to investigate the influence of pretreatment NLR and PCT on progression-free survival (PFS) in extensive-stage SCLC patients treated with first-line chemotherapy.

## Materials and methods

### Patients

A total of 100 patients who had been diagnosed with extensive-stage SCLC histopathologically and clinically at Shanghai Chest Hospital from February 2018 to February 2019 were enrolled in our retrospective study. The follow-up ended on October 15^th^, 2019.

The study protocol was approved by the Ethics Committee of Shanghai Chest Hospital and conducted in accordance with the Declaration of Helsinki (as revised in Fortaleza, Brazil, October 2013). Due to the retrospective nature of this study, the need for informed consent was waived.

The key inclusion criteria were as follows: (1) patients whose data on pretreatment NLR and PCT levels were available; (2) patients without any kind of infections at diagnosis or during treatment; (3) patients who had at least one measurable tumor lesion; (4) patients who aged between 18 and 70; (5) patients whose Eastern Cooperative Oncology Group performance status were 0 to 1; (6) patients who received cisplatin or carboplatin combined with etoposide as first-line chemotherapy.

### Data collection

The clinical characteristics included gender, age, smoking history, anatomy type, response to first-line chemotherapy, pretreatment NLR and PCT levels. All enrolled patients received response evaluations every two courses of chemotherapy according to the Response Evaluation Criteria in Solid Tumors (RECIST v1.1). The PFS was defined as the time from the initiation of first-line chemotherapy to disease progression, death or the last follow-up visit. Data on pretreatment NLR and PCT levels were collected at the initiation of first-line chemotherapy. The NLR was defined as the absolute neutrophil count divided by the absolute lymphocyte count. The normal reference value of PCT in our study ranged from 0 to 0.05 ng/ml.

### Statistical analysis

Patients were stratified according to the median values of pretreatment NLR and PCT levels. SPSS22.0 statistical software (IBM, Armonk, NY, USA) was used to analyze data. The Chi-squared test was used to identify the clinical characteristics

related to pretreatment NLR (or PCT). The Kaplan-Meier method and log-rank test were used to investigate the association between pretreatment NLR (or PCT) and PFS. The multivariable Cox regression model was used to screen out the significant factors related to PFS. All analyses were two-sided, and *p* values ＜0.05 were defined as statistical significance.

## Results

### Patient characteristics

Our retrospective study included 100 extensive-stage SCLC patients. The male and female patients accounted for 86% and 14%, respectively. Of the total patients, 84(84%) patients were ever-smokers while 16(16%) patients were never-smokers. Central type(73%) was the main anatomy type of all patients. In terms of response to first-line chemotherapy, 60(60%), 30(30%) and 10(10%) patients achieved partial response (PR), stable disease(SD) and progressive disease(PD), respectively. The median value of pretreatment NLR level was 3.17 (range:1.0–10.1). The median value of pretreatment PCT level was 0.06 (range:0.02–1.49; ng/ml) (Table 1).

**Table 1.** Clinical characteristics of the 100 patients.

Characteristic	N(%)
Gender	
Male/Female	86(86%)/14(14%)
Age(years)	
≤65/＞65	57(57%)/43(43%)
Smoking history	
No/Yes	16(16%)/84(84%)
Anatomy type	
Central type/Peripheral type	73(73%)/27(27%)
Response to first-line chemotherapy	
PD/SD/PR	10(10%)/30(30%)/60(60%)
NLR (range:1.0–10.1; median: 3.17)	
≤3.17/＞3.17	50(50%)/50(50%)
PCT (range:0.02–1.49; median:0.06; ng/ml)	
≤0.06/＞0.06	52(52%)/48(48%)

PD: progressive disease; SD: stable disease; PR: partial response: NLR: neutrophil-to-lymphocyte ratio; PCT: procalcitonin.

### The association between pretreatment NLR (or PCT) and clinical characteristics

The statistical results showed that pretreatment NLR or PCT was not significantly associated with gender, age, smoking history, anatomy type, and response to first-line chemotherapy (Tables 2, 3).

**Table 2.** The association between pretreatment NLR and clinical characteristics.

Characteristic	NLR ≤3.17(*n* = 50)	NLR＞3.17(*n* = 50)	P-value
Gender			
Male	46(92%)	40(80%)	0.084
Female	4(8%)	10(20%)	
Age(years)			
≤65	28(56%)	29(58%)	0.840
＞65	22(44%)	21(42%)	
Smoking history			
No	7(14%)	9(18%)	0.585
Yes	43(86%)	41(82%)	
Anatomy type			
Central type	40(80%)	33(66%)	0.115
Peripheral type	10(20%)	17(34%)	
Response to first-line chemotherapy			
PD	4(8%)	6(12%)	0.465
SD	13(26%)	17(34%)	
PR	33(66%)	27(54%)	

PD: progressive disease; SD: stable disease; PR: partial response: NLR: neutrophil-to-lymphocyte ratio.

**Table 3.** The association between pretreatment PCT and clinical characteristics.

Characteristic	PCT ≤0.06 (*n* = 52)	PCT＞0.06(*n* = 48)	P-value
Gender			
Male	47(90%)	39(81%)	0.188
Female	5(10%)	9(19%)	
Age(years)			
≤65	30(58%)	27(56%)	0.884
＞65	22(42%)	21(44%)	
Smoking history			
No	7(14%)	9(19%)	0.471
Yes	45(86%)	39(81%)	
Anatomy type			
Central type	34(65%)	39(81%)	0.074
Peripheral type	18(35%)	9(19%)	
Response to first-line chemotherapy			
PD	6(11%)	4(8%)	0.829
SD	16(31%)	14(29%)	
PR	30(58%)	30(63%)	

PD: progressive disease; SD: stable disease; PR: partial response; PCT: procalcitonin.

### Progression-free survival

**Figure 1.**
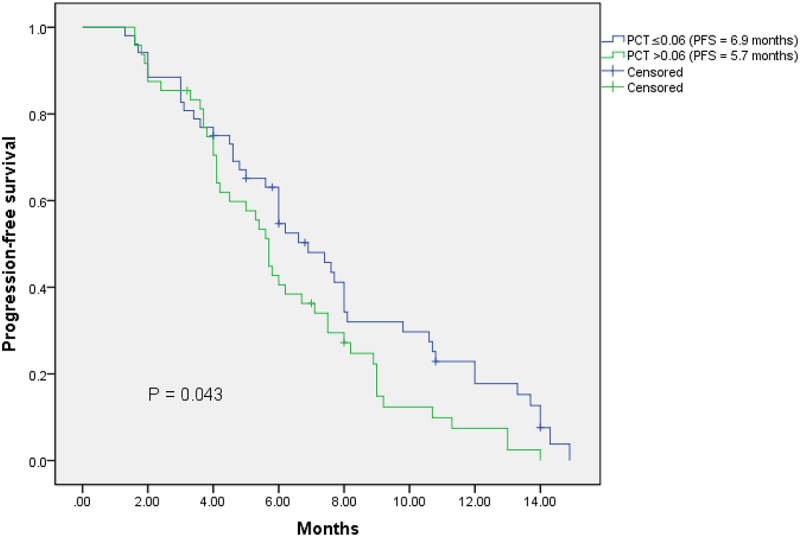
Kaplan – Meier analysis of the 100 SCLC patients according to the stratification of pretreatment PCT.

**Figure 2.**
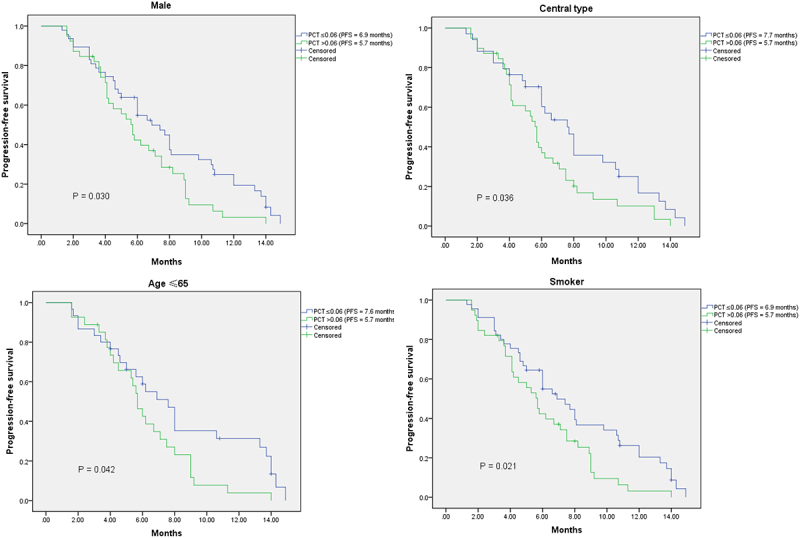
Kaplan – Meier analysis of subgroups according to the stratification of pretreatment PCT.

The median PFS of the total extensive-stage SCLC patients was 6.0 months. The median PFS of male and female patients were 6.0 months and 5.7 months, respectively (*p* = .448). The median PFS of patients with smoking history and patients without smoking history were 6.0 months and 5.7 months, respectively (*p* = .368). The median PFS of low pretreatment NLR group (NLR ≤3.17) was longer than that of high pretreatment NLR group (6.2 months vs 5.8 months), however, no significant difference was found (*p* = .675). Most importantly, our study showed that patients with low pretreatment PCT (PCT ≤0.06; ng/ml) had significantly better PFS than patients with high pretreatment PCT (PCT＞0.06; ng/ml) (6.9 months vs 5.7 months; *p* = .043)(Figure 1). The same results were observed in the male subgroup (*p* = .030), central type subgroup (*p* = .036), age (≤65) subgroup (*p* = .042), and smoker subgroup (*p* = .021) (Figure 2). With the multivariable Cox regression analysis, the response to first-line chemotherapy (*p* ≤ .001) and pretreatment PCT (HR = 0.516; 95%CI 0.326–0.817; *p* = .005) were identified as independent factors associated with PFS (Table 4).

**PFS**: progression-free survival; PCT: procalcitonin

**PFS**: progression-free survival; PCT: procalcitonin

**Table 4.** Univariate and multivariate analyses with regard to PFS.

Characteristic	Median PFS (months)	Univariate analysis (P-value)		Multivariate analysis [HR (95%CI)]	P-value
Gender					
Male	6.0		0.448		
Female	5.7				
Age(years)					
≤65	6.2		0.210		
＞65	6.0				
Smoking history					
No	5.7		0.368		
Yes	6.0				
Anatomy type					
Central type	6.0		0.915		
Peripheral type	6.0				
Response to first-line chemotherapy					
PD	1.7		≤0.001		≤0.001
SD	6.0				
SD	6.0		0.056		
PR	6.9				
NLR					
≤3.17	6.2		0.675		
＞3.17	5.8				
PCT (ng/ml)					
≤0.06	6.9		0.043	0.516(0.326–0.817)	0.005
＞0.06	5.7				

PD: progressive disease; SD: stable disease; PR: partial response: NLR: neutrophil-to-lymphocyte ratio; PCT: procalcitonin; PFS: progression-free survival; HR: hazard ratio; CI: confidence interval.

## Discussion

Our retrospective study investigated the prognostic value of pretreatment NLR and PCT in extensive-stage SCLC patients treated with first-line chemotherapy. To our knowledge, there are few studies about the relationship between pretreatment PCT and survival in extensive-stage SCLC patients. This study shows lower pretreatment PCT is associated with better PFS in extensive-stage SCLC patients treated with first-line chemotherapy.

NLR reflects the balance between neutrophils and lymphocytes, which is a biomarker of systemic inflammatory response. Previous studies have shown that high levels of neutrophils promote cancer cell proliferation, invasion, metastasis, and induce resistance to cancer therapeutics; high levels of lymphocytes inhibit tumor growth and invasion through anti-cancer immune response. The implications of NLR have been discussed in previous SCLC-related studies. A retrospective study of 139 SCLC patients showed that high NLR was associated with poor OS.^12^ Ryoko et.al reported that higher pretreatment NLR predicted worse OS in extensive-stage SCLC patients.^13^ Another study indicated that NLR could be used as a negative prognostic factor for OS in extensive-stage SCLC patients.^5^ Overall, these findings imply that NLR may predict the long-term survival of SCLC. Besides, a research on extensive-stage SCLC showed that the PFS of first-line chemotherapy was significantly different according to the stratification of baseline NLR, and patients with NLR＜3 could have PFS benefit.^5^ Kang et al. also reported some similar results.^14^ Inflammatory responses to tumors are mediated by the release from neutrophils of inflammatory cytokines, leukocytic and other phagocytic mediators that induce damage to cellular DNA, inhibit apoptosis and promote cancer-associated angiogenesis.^15^ A higher NLR indicates a heavier tumor burden and higher level of inflammation, which may be associated with a poor prognosis. However, our study found no significant relationship between pretreatment NLR and PFS in extensive-stage SCLC patients, though the median PFS of low NLR group was a bit longer compared with high NLR group. This may be explained by the fact that some extensive-stage SCLC patients are quite sensitive to chemotherapy despite of the heavy tumor load. Recently, immunochemotherapy based on atezolizumab and durvalumab has been introduced into treatment of SCLC. Few researchers also evaluated the prognostic significance of NLR in extensive-stage SCLC patients treated with immunochemotherapy. For example, Satoshi et al. found no significant relationship between NLR and prognosis in 16 extensive-stage SCLC patients treated with first-line atezolizumab plus carboplatin and etoposide therapy.^16^ However, Yasin et al. found that NLR was associated with poor prognosis in 55 extensive-stage SCLC patients who received front-line atezolizumab with etoposide plus platin regimen.^17^ The difference of two studies might be due to sample size.

PCT is widely used to guide the diagnosis and treatment of bacterial infection. Unlike NLR, we did not know much about the implications of PCT in lung cancer. PCT is secreted by the C-cell of thyroid and neuroendocrine cells located in lung, adrenal, liver, kidney, and muscles.^18^ Biosynthesis of PCT in small cell carcinomas of the lung was firstly reported by Cate et.al,^8^ then, neuroendocrine cancers including SCLC are known to be associated with PCT elevation. A previous study showed that pretreatment PCT level above 0.15 ng/ml was significantly associated with shorter OS in lung cancer patients.^7^ In our study, extensive-stage SCLC patients with pretreatment PCT level under 0.06 ng/ml could have PFS benefits after first-line chemotherapy. Our results may be explained by the fact that a greater PCT elevation in SCLC patients is associated with a heavier tumor burden especially in extensive stage, which leads to poorer prognosis. Further research is needed to determine the specificity of PCT in SCLC patients and particularly explore the potential usefulness of PCT to monitor the response to chemotherapy.

Our study also has some limitations. Firstly, it is a retrospective study: secondly, the data of OS are not available due to the high rate of lost to follow-up.

To sum up, we find that pretreatment PCT is an independent factor associated with PFS in extensive-stage SCLC patients treated with first-line chemotherapy, however, pretreatment NLR reflects no significant prognostic value in our study.

## Data Availability

The data used to support the findings of this study are available from the first author and the corresponding author upon request.
